# Arthroscopic-assisted reduction for Developmental Hip Dysplasia (DDH) through the sub-adductor and anterolateral portals; A 24-month follow-up prospective descriptive study

**DOI:** 10.1186/s12891-024-08234-y

**Published:** 2025-01-08

**Authors:** Amr Samir Rashwan, Mahmoud El-Desouky, Hassan Elbarbary, Mahmoud Abd Elhamid Madbouly, Ahmed Khedr

**Affiliations:** Department of Trauma and Orthopedics, Faculty of Medicine, Cairo University, Giza, Egypt

**Keywords:** DDH, Arthroscopic reduction, Open reduction, Hip arthroscopy

## Abstract

**Background:**

Developmental dysplasia of the hip (DDH) encompasses a spectrum of pathological conditions, including dislocation, subluxation, and deformities of the femoral head and acetabulum. The optimal surgical approach for DDH remains a subject of debate. Successful treatment aims to achieve a stable concentric reduction and prevent future subluxation or dislocation. This study aims to assess the clinical and radiographic outcomes of arthroscopic-assisted surgical reduction of DDH in children aged from 6 months to 5 years old.

**Methods:**

This prospective study included 57 patients with DDH (68 involved hips) between January 2019 and May 2021. They were treated with arthroscopic-assisted surgical reduction. Dega osteotomy was needed in 22 cases, femoral osteotomy and or shortening was necessary in 11 cases, and combined pelvic and femoral osteotomies were required in nine cases. We evaluated and followed all the patients clinically and radiologically, using Severin, modified Severin scores, Shenton line, and acetabular index measurement up to 24 months postoperatively.

**Results:**

The mean age of the included patients was 26.9 months. The mean operative time was 54.7 (36–90) minutes. Clinical assessment using the modified Severin classification revealed that 53 hips (77.9%) were grade I and 11 hips (16.2%) were grade II at the end of the follow-up. Radiological evaluation using Severin classification revealed that 55 hips (80.9%) were in grade I, and 10 hips (14.7%) were in grade II. There was a statistically significant correlation between clinical and radiological grading (*p* < 0.001). Hip re-dislocation and avascular necrosis (AVN) were experienced in one and two cases, respectively.

**Conclusion:**

These findings suggest that arthroscopic-assisted reduction for DDH, with or without osteotomies, is a promising technique with satisfactory clinical and radiographic outcomes and a low complication rate. However, given the single-center nature of this study and its relatively small sample size, these results should be interpreted with caution.

**Clinical Trial Registration (Retrospectively registered):**

Registration number: NCT06520436. 25-7-2024.

## Introduction

Developmental dysplasia of the hip (DDH) involves a broad range of pathologic changes affecting the developing hip. These changes range from acetabular dysplasia to hip subluxation or dislocation [1]. DDH is the most common disorder of the hip in children [2, 3]. Ten out of 1,000 live births (1%) have either hip subluxation or dysplasia. One out of 1,000 live births (0.1%) has a dislocatable hip [4, 5].

The ideal treatment for DDH is still debated across various age groups. While surgical reduction without osteotomy aims to minimize invasiveness, and preserve the hip joint, residual instability often necessitates peri-acetabular and sometimes even femoral osteotomies to achieve and maintain a stable reduction. Evidence suggests that the choice between these approaches depends on patient age, severity of dislocation, and associated anatomical deformities [6].

The open reduction procedures carry a high incidence of complications ranging from hip stiffness, flexion deformity, subluxation, residual acetabular dysplasia, and re-dislocation [7–9]. Also, the capsular incision carries a major risk for avascular necrosis (AVN) [10–12].

The arthroscopic approach carries the merits of avoiding the hazards of open procedures [13, 14]. Using combined anterolateral and sub-adductor portals offers better visualization of the acetabular cavity and instrumentation during addressing pulvinar tissue, ligamentum teres, and transverse acetabular ligament (TAL) [15].

This study aims to evaluate the clinical and radiographic outcomes of arthroscopic-assisted surgical reduction with or without osteotomy in the management of children diagnosed with developmental dysplasia of the hip (DDH) after 24 months of follow-up.

## Methods

We conducted this prospective descriptive clinical trial with the approval of the Research Ethics Committee (REC). From January 2019 to May 2021, we enrolled in this study 60 patients aged from 6 months to 5 years old who had been diagnosed with developmental dysplasia of the hip (DDH) and needed to undergo surgery. We included patients who had failed trials of closed reduction. Children who had previously undergone surgery for DDH or had teratologic or neuromuscular dislocations were excluded. Convenience sampling was utilized to enroll participants in the study, whereby patients were chosen based on their availability and eligibility according to specific inclusion and exclusion criteria.

**Fig. 1:**
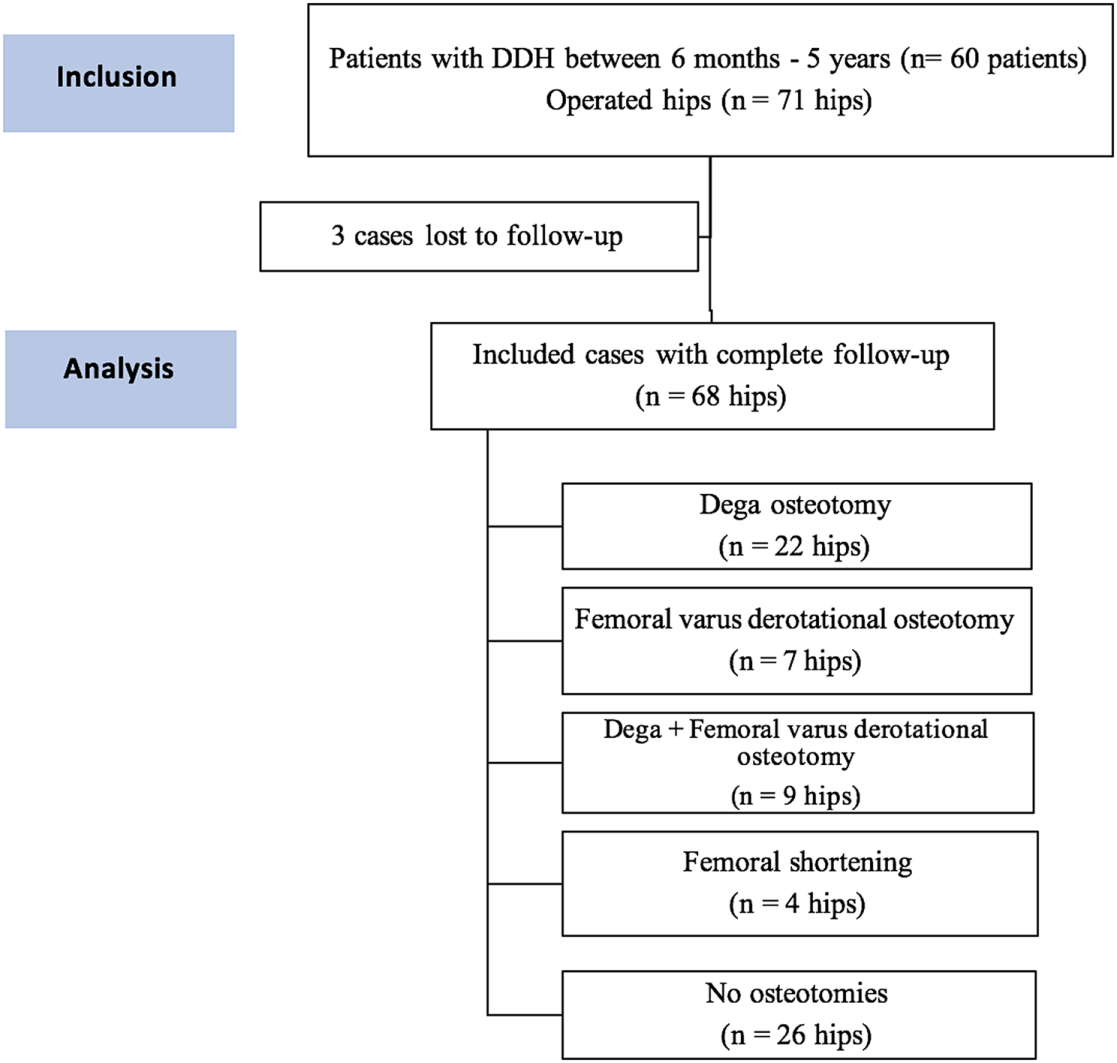
Flow chart of included cases

Throughout this trial, we operated on 11 patients on both sides, resulting in the inclusion of 71 affected hips. At the end of the study, 57 patients, with 68 affected hips, had completed 24 months of follow-up (Fig. 1).

We evaluated the included patients by taking a detailed history from the parents, conducting a thorough clinical examination, and using pelvis X-rays to measure the acetabular index (AI), align the Shenton line, and evaluate the size of the femoral head ossific center. We used Tönnis classification to radiographically assess the degree of dislocation in all hips in this study [16].

Arthroscopic-assisted reduction was used as a one-stage operative treatment for all hips. In addition to that, Dega pelvic osteotomy and/or femoral shortening and varus derotation osteotomies were performed when necessary.

### Surgical procedure

A challenging part of the procedure is placing the portals without injuring the neurovascular structures around the hip joint. What adds to the complexity is that the surface anatomy is distorted as the greater trochanter and the head of femur positions are variable according to the Tönnis classification.

**Fig. 2:**
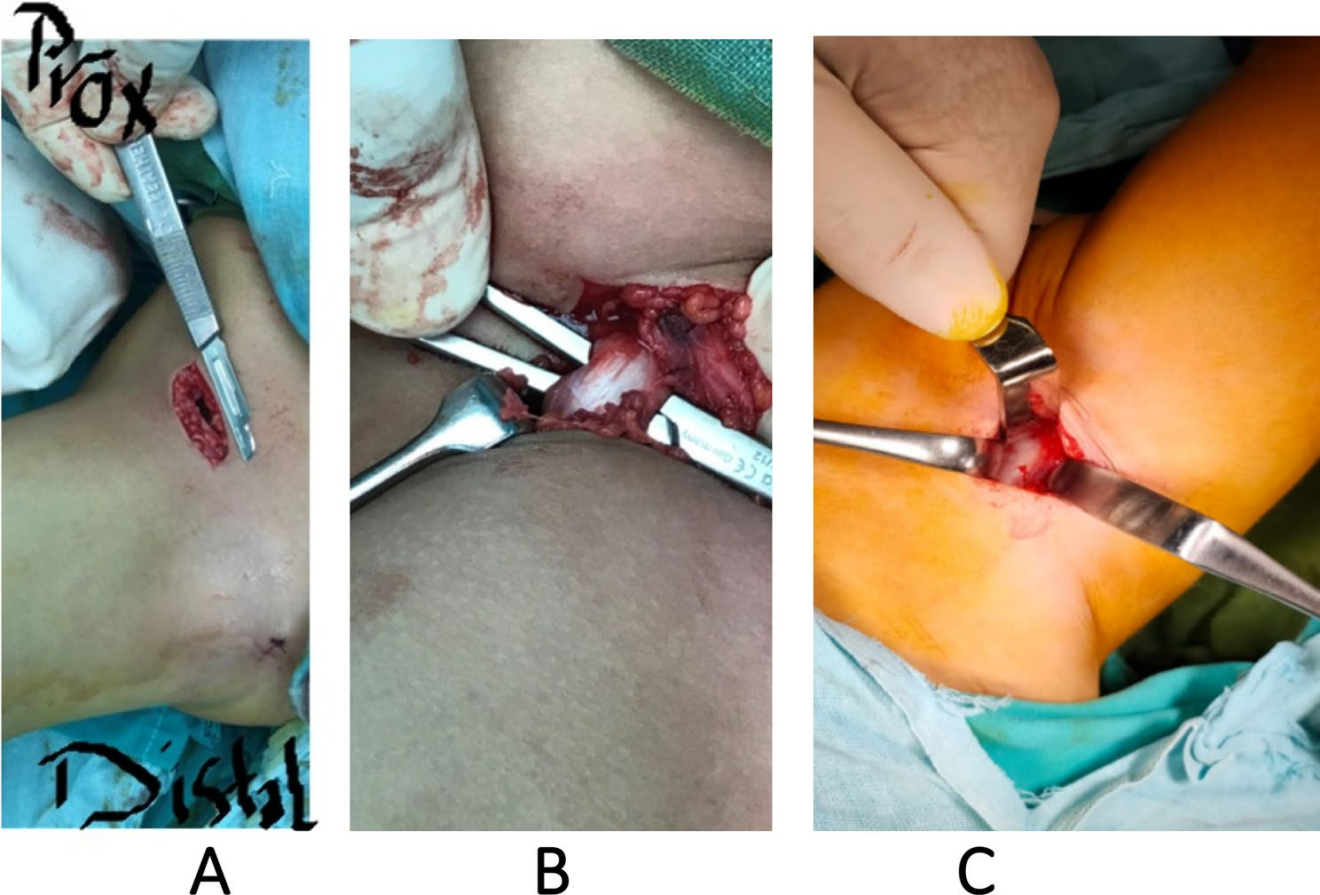
**A**: Skin incision from ASIS distally into the thigh for about 3 to 4 cm, **B**: Recession of the iliopsoas tendon **C**: The front of the capsule of the hip joint is exposed

While the patients were positioned supine on a radiolucent table, the procedure began with a percutaneous adductor tenotomy. We abducted the hip and palpated the tight adductor longus under the skin. A 15-blade was introduced to cut the tendon. We later used this wound to develop the sub-adductor portal. The next step was to perform the iliopsoas release. Approximately 3 to 4 cm of skin incision was extended from the anterior superior iliac spine (ASIS) distally into the thigh (Fig. 2). The skin incision was approximately one-third the size of the standard anterolateral incision. Lateral femoral cutaneous nerve was identified and medially retracted, followed by fascia lata incision. To facilitate the opening of the interval between the tensor fascia lata muscle laterally and the sartorius muscle with the rectus femoris muscle medially, blunt dissection was used. The hip joint capsule’s anterior portion was subsequently exposed. To perform recession, the iliopsoas tendon was identified and grasped using right-angle forceps (Fig. 2).

The hip was internally rotated to move the femoral head posteriorly, and a pick-up was used to pull the capsule. We then created the anterolateral portal by making an incision directly into the capsule at the inferomedial aspect of the head and introduced a 2.7-mm, 30-degree scope through it to visualize the femoral head. We used an arthroscopic pump and the pressure was inflated to 30 mmHg. The hypertrophied ligamentum teres was seen by rotating the hip internally and externally and then followed till visualizing the transverse acetabular ligament (TAL) and the acetabulum. A needle was introduced through the incision used for the adductor tenotomy in a cranial and anterior direction to be visualized inside the hip joint. The capsule was pierced by a straight hemostat placed just anterior to the needle to be visualized into the joint, thus developing the sub-adductor portal, which was used for instrumentation.

**Fig. 3:**
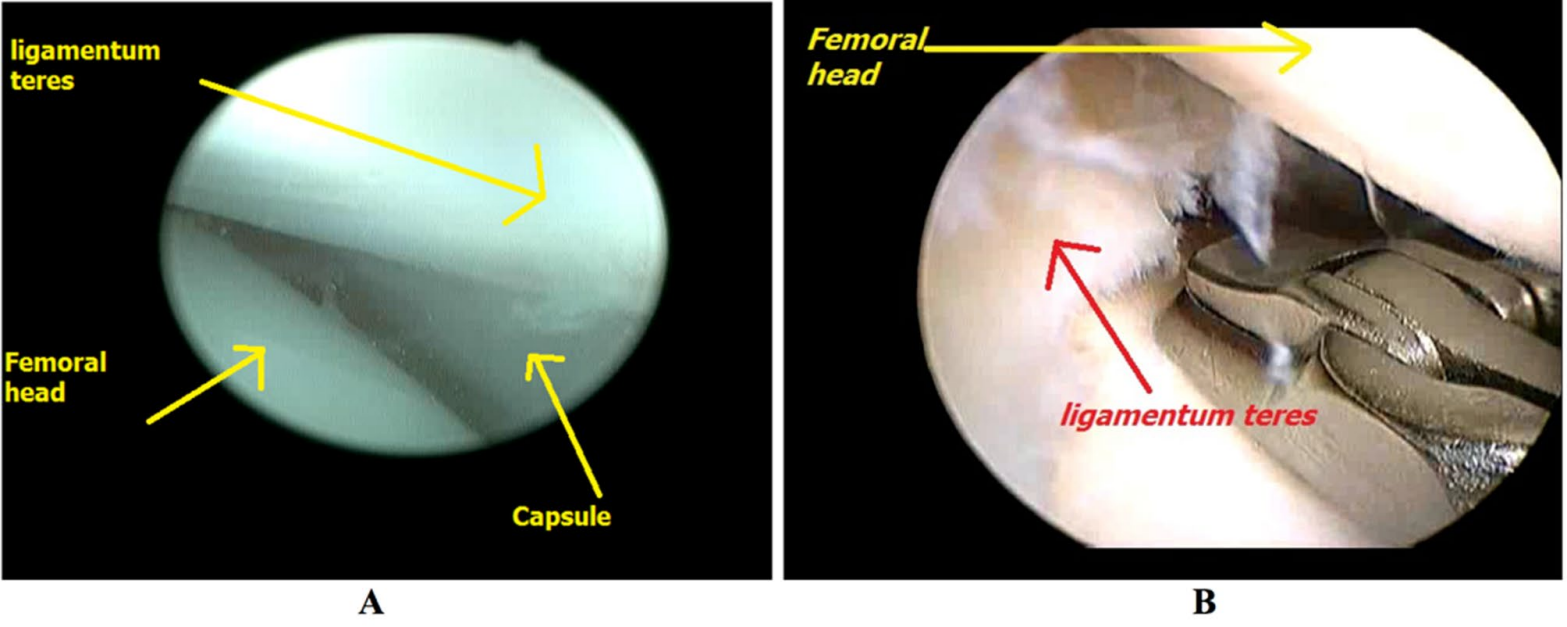
**A**: Visualization of ligamentum teres, **B**: Cutting ligamentum teres from its femoral attachment

The ligamnetum teres was followed and visualized as close as possible to its femoral attachment, which may be aided by doing some external and internal rotation of the hip. Another useful maneuver could be performed by the assistant by stabilizing the hip with one hand and doing hip abduction with distraction of the femur to create more working space. The ligmaenum teres was then cut using a basket introduced through the sub-adductor portal (Fig. 3).

Switching the portal was the next step, using the sub-adductor portal as a viewing portal for better visualization of the acetabulum after cleaning the pulvinar tissue using either a shaver or electrocautery. The latter was preferred as pulvinar is a densely vascularized fibrofatty tissue that may cause extensive bleeding.

**Fig. 4:**
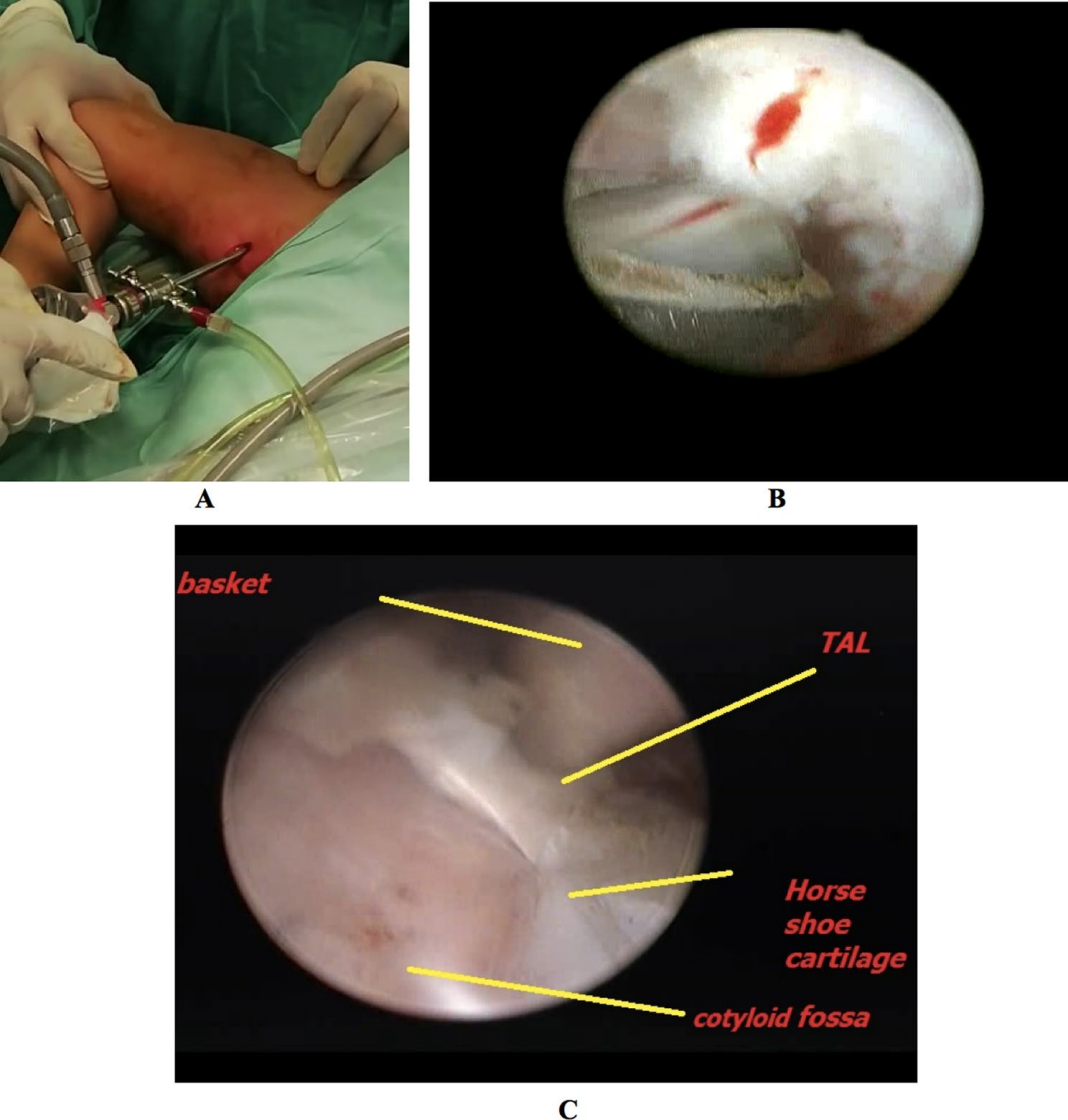
**A**: Using the sub-adductor portal as a viewing portal, **B**: Shaving the pulvinar, **C**: Cutting the transverse acetabular ligament (TAL)

While still using the same portals, an arthroscopic hook was introduced now through the sub-adductor portal. The ligamentum teres was followed proximally to the direction of the acetabulum and used as a guide to the TAL. The TAL was identified by sliding the hook along the acetabulum until it fell into the acetabular notch inferiorly. The basket was then introduced to cut the TAL anterior and posterior to the attachment of the ligamentum teres (Fig. 4). The ligamentum teres with the TAL were extracted by a grasper through the sub-adductor portal out of the hip joint. The hook was introduced once again to confirm that the TAL was adequately released. Now the hook should follow the acetabulum until it falls into the acetabular notch. If the hook was pulled, there should not be any soft tissue resistance, ensuring successful TAL release.

**Fig. 5:**
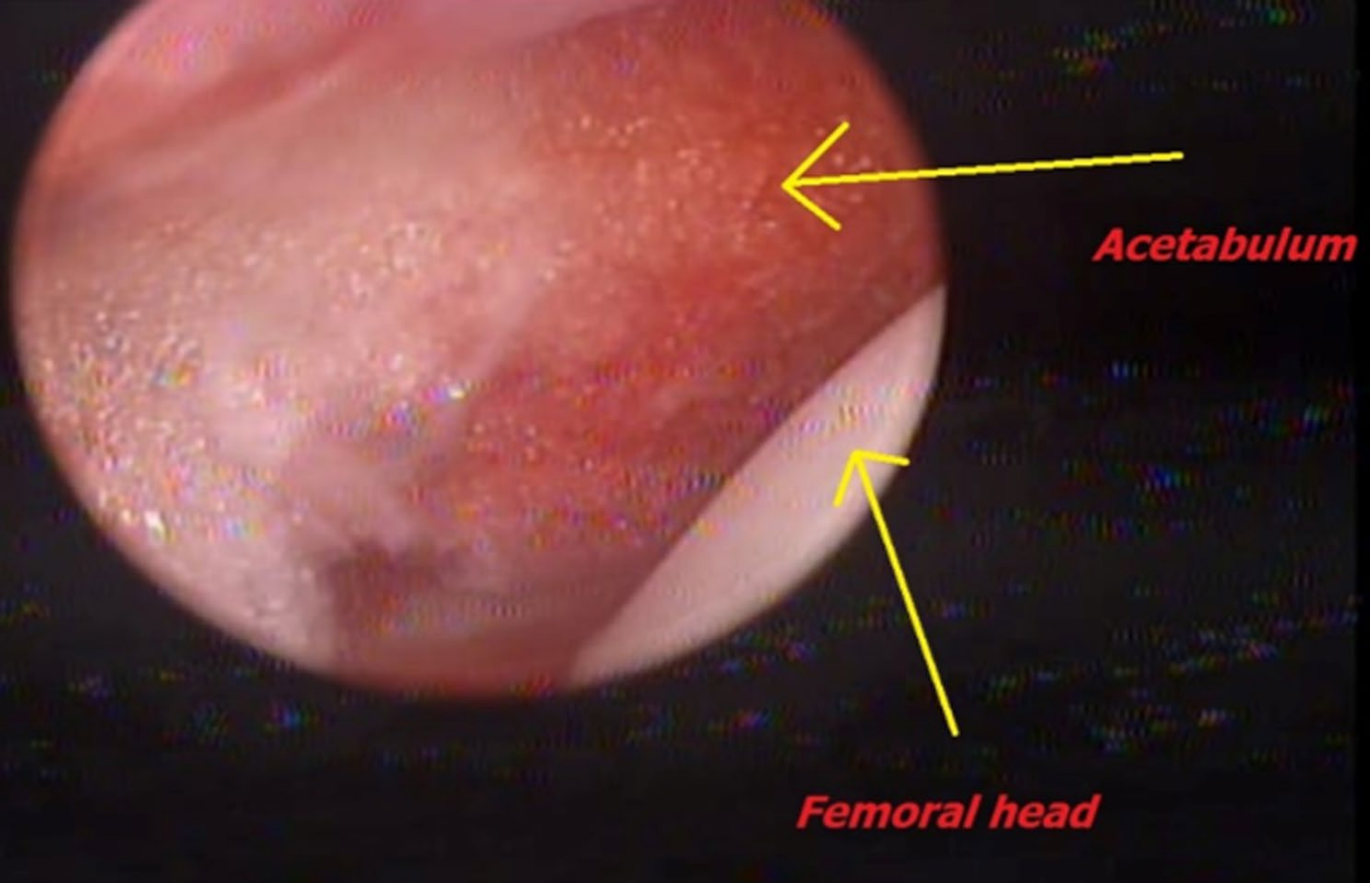
Femoral head can be seen as it enters the acetabulum through the sub-adductor portal

To achieve femoral head reduction, the hip should be flexed, abducted, and medially rotated while simultaneously applying traction and pressure. Excision of the labrum was not a constant step in our procedure. In cases when reduction was difficult to achieve after resection of ligamentum teres and TAL and after excision of pulvinar tissue, the abnormally positioned labrum was either retracted or excised. The reduction was assessed arthroscopically and radiologically by C-arm (Fig. 5). We evaluated the stability and necessity of additional femoral or pelvic osteotomy, or both, in hips where the femoral head could be reduced. If the hip remained stable during internal rotation and abduction, a femoral varus derotational osteotomy was performed. On the other hand, we performed Dega innominate osteotomy, if the hip exhibited stability in flexion and abduction. Moreover, a combination of femoral derotational and varus osteotomy, along with Dega osteotomy, was performed, if the hip demonstrated stability in flexion, internal rotation, and abduction. If the femoral head was repositioned under tension while the leg was in a neutral position, this indicated the need for a simultaneous femoral shortening osteotomy. Femoral osteotomy was done by the technique described by Wenger et al., and Dega osteotomy was done by the technique described by Grudziak et al. [17, 18]. For the Dega osteotomy, a 3–5 cm skin incision was put over the anterior part of the iliac crest. The capsule of the hip joint was not opened. At the end of the procedure, the wounds were closed in the standard fashion.

### Postoperative care

The patients were placed in a hip spica cast for 12 weeks, with the hip joint positioned at a 40-degree abduction, 60-degree flexion, and neutral rotation. No specific rehabilitation protocol was followed after cast removal. Parents were instructed to encourage their children to start weight-bearing, and active mobilization and to return to normal activities at their own pace. No formal physical therapy sessions were prescribed. We performed routine X-rays at six weeks, three, six, 12, and 24 months postoperatively to evaluate head containment, acetabular index regression, osteotomy healing, head ossific center development, and any radiological evidence of AVN of the femoral head. Patients were clinically and radiographically evaluated by modified Severin and radiographic Severin classifications [19, 20]. All clinical and radiographic measurements were carried out by a qualified single assessor; the fourth author M.A.M (M.D.).

### Statistical methods

We processed the data using SPSS 28. Data were summarized using the mean and standard deviation or count and percentages. The normality of the sampling distribution was assumed based on the Central Limit Theorem. Paired-t-test was used to compare the acetabular index before and after the procedure at the 24-month follow-up measurements. Statistically significant results were defined as having p-values less than 0.05.

Post-hoc power analysis was done using the R package pwr (version 1.3.0) within R software (version 4.3.1). We utilized the observed means and standard deviations of the acetabular index before and after the procedure (Mean was 42.84^o^ ± 5.33^o^ before the operation and 20.07^o^ ± 4.74^o^ afterward) and the total sample size of 68 hips. The significance level (α) was set at 0.05, and a two-tailed paired t-test was assumed.

## Results

The mean age of the included cases was 26.9 ± 5.5 months, with a range of 9 to 53 months. The average duration of the operation was 54.7 min with a range between 36 and 90 min. (Table 1) presents the demographic information of the cases involved, their pre-operative radiographic grading based on the Tönnis classification, and their post-operative clinical and radiographic grading.

**Table 1:** Demographic features, pre-operative measurements, and post-operative grading of included cases

		Total	
		Count	%
Sex	Male	9	15.8%
	Female	48	84.2%
Age group	6–24 months	30	52.6%
	24–36 months	15	26.3%
	> 36 months	12	21.1%
Side	Right	17	29.8%
	Left	29	50.9%
	Bilateral	11	19.3%
Pre-operative degree of dislocation (Tonnis classification)	Grade II	6	8.8%
	Grade III	19	27.9%
	Grade IV	43	63.2%
Osteotomies	Dega osteotomy	22	32.4%
	Femoral varus derotational osteotomy	7	10.3%
	Dega + Femoral varus derotational osteotomies	9	13.2%
	Femoral shortening osteotomy	4	5.9%
Clinical outcome (modified Severin classification)	Grade I	53	77.9%
	Grade II	11	16.2%
	Grade III	4	5.9%
Radiological evaluation (Severin classification)	Grade I	55	80.9%
	Grade II	10	14.7%
	Grade III	3	4.4%
	Grade IV	0	0.0%
	Grade V	0	0.0%
	Grade VI	0	0.0%
Complications	Re-dislocation	1	1.5%
	Persistent subluxation	3	4.4%
	AVN	2	2.9%
	Sciatic nerve neurapraxia	1	1.5%

Upon comparing the X-rays taken before the surgery and the most recent ones, there was a statistically significant reduction in the acetabular index (95% CI: 21.40-24.13, *p* < 0.001), with a mean value of 42.84^o^ ± 5.33^o^ before the operation and 20.07^o^ ± 4.74^o^ afterward.

Regarding postoperative complications, only one case (1.5%) experienced hip re-dislocation within the first 3 months after surgery. The 28-month-old child had a good acetabular index as the Dega osteotomy was initially performed. Therefore, the revision surgery involved open reduction and capsulorrhaphy.

Three hips (4.4%) had persistent subluxation, and they were managed by nighttime hip abduction brace for 5–6 months at night only. AVN and temporary sciatic nerve neurapraxia were seen in two cases (2.9%) and one case (1.5%), respectively (Table 1). There were no cases with stiffness; that’s why physiotherapy was not needed. None of the operated cases experienced infection.

## Discussion

Developmental Dysplasia of the Hip (DDH) encompasses a range of hip abnormalities in newborns, from acetabular dysplasia to irreducible hip dislocation. The ideal treatment for DDH is still debated across various age groups. Both closed and open reduction methods are used, with or without pelvis and/or femoral osteotomies. However, a high incidence of complications ranging from hip stiffness, flexion deformity, subluxation, re-dislocation, residual hip dysplasia, and AVN can occur regardless of the method of treatment [1, 7–9].

High AVN rates are inevitable due to femoral head injury hazards. Additionally, there is a considerable risk of AVN with the extensive incision in the joint capsule [10–12].

Our case series included 68 hips of 57 patients with DDH aged 9–53 months, with a mean age of 26.9 months, who underwent arthroscopic-assisted surgical reduction with or without pelvic or femoral osteotomy and were followed up clinically and radiologically for at least 24 months.

The study found satisfactory (Grade I, II) results in 64 hips (94.1%) and unsatisfactory (Grade III) results in four hips (5.9%) using modified Severin grading for clinical evaluation. Similarly, the radiological results were satisfactory (Grade I, II) in 65 hips (95.6%) and unsatisfactory (Grade III to VI) in three hips (4.4%). There was a significant improvement when comparing the acetabular index pre and postoperatively. During follow-up, one patient (1.5%) encountered re-dislocation, while two cases (2.9%) faced AVN at the final evaluation.

One of the interesting findings is that patients operated by arthroscopic technique had minimal stiffness and good hip range of motion (ROM) immediately after removing the cast. We did not systemically record the postoperative ROM after removing the cast because this was not included in the initial study protocol. However, we had this impression from the patient cohort.

Although capsulorrhaphy was previously considered a standard and main step performed during open reduction of DDH cases; recently the importance and value of this step is questionable. In recent literature where open reduction without capsulorrhaphy was done, did not result in higher re-dislocation rates [21, 22]. Moreover, in DDH cases openly reduced through the medial approach, capsulorrhaphy is not performed which supports that open reduction can be performed without the need for capsulorrhaphy [23–25].

The arthroscopic appearance of dislocated hips was initially described by Gross in 1977. He examined four hips using a 2.2 mm needlescope, combining anterior and medial approaches under the adductor longus tendon. Although the hip arthroscopy was interesting as he stated, he did not add to current knowledge [26].

Later studies demonstrated promising results of the arthroscopic-assisted approach. In several studies, successful reduction of dislocated hips has been reported alongside with significant reduction in the postoperative acetabular index and low AVN and re-dislocation rates (Table 2). Although in most of these studies, a few number of cases have been investigated, Xu et al.‘s study on a larger cohort of 35 cases (40 hips) and longer mean follow-up period (71 months) showed consistent results. They also reported a shorter mean operative time (28 min) compared with previous studies and our series in which the mean operative time was 55 min. We owe this to the need for additional pelvic and/ or femoral osteotomies in a large number of cases; 42 hips (61.8%) which were performed in the same session [15, 27–30].

**Table 2:** Comparison of the data of arthroscopic-assisted reduction studies

Study	Procedure	Sample size		Age (months)		Follow-up (months)		Operative time (minutes)		Acetabular index (^o^)		Clinical grading	Radiographic grading	Complications		
		Cases (n)	Hips (n)	Mean	Range	Mean	Range	Mean	Range	Pre	Post			AVN	Re-dislocation	Other complications
Bulut et al. [27]	Arthroscopic-assisted reduction through anterolateral & anteromedial portals	4	4	12.8	11–14	13.7	12–16			40.3	29			NR	NR	NR
McCarthy et al. [28]	Arthroscopic-assisted reduction through anterolateral & posterolateral portals	3	3	14		9				35	30			1 (33.3%)	NR	Residual dysplasia in one hip (33.3%)
Eberhardt et al. [15]	Arthroscopic-assisted reduction through anterolateral & sub-adductor portals	5	8	5.8	4–7	13.2	9–24			35.5 (30–40)	23.3 (17–28)			3 (37.5%)	NR	NR
Öztürk et al. [29]	Arthroscopic-assisted reduction through anterolateral & anteromedial portals	9	9	13.1	9–16	47.7	22–79	40	35–65	39.9 (34–52)	26 (22–34)			1 (11.1%)	NR	Residual dysplasia in 2 hips (22.2%)
Xu et al. [30]	Arthroscopic-assisted reduction through anterior & anterosuperior greater trochanter portals +/- Pemberton & Femoral osteotomies	35	44	17.7	4–40	71	36–96	28	22–36	43.8 (31–55)	29.5 (22–41)	*McKay grading* Excellent = 35 Good = 9 Fine = 0 Poor = 0	*Severin grading* Grade I = 27 Grade II = 10 Grade III = 4 Grade IV = 3 Grade V = 0 Grade VI = 0	4 (9.1%)	3 (6.8%)	NR
Our Study	Arthroscopic assisted reduction through anterolateral & sub-adductor portals +/- Dega & Femoral osteotomies	57	68	26.9	9–53	24		54.7	36–90	42.84 ± 5.33	20.07 ± 4.74	*Modified Severin grading* Grade I = 53 Grade II = 11 Grade III = 4	*Severin grading* Grade I = 55 Grade II = 10 Grade III 3 Grade IV = 0 Grade V = 0 Grade VI = 0	2 (2.9%)	1 (1.5%)	Persistent subluxation in 3 hips (4.4%) Sciatic nerve neurapraxia in 1 hip (1.5%)

NR: Not Reported

While Eberhardt et al. used the same portals we used, but they didn’t switch portals as they used the sub-adductor portal as a viewing portal while the procedure was performed through the anterolateral portal. In our procedure, we resected the femoral attachment of ligamentum teres through the sub-adductor portal while its acetabular attachment, TAL, and pulvinar tissue were removed through the anterolateral portal [15].

In contrast, studies investigating the traditional open reduction techniques whether through the anterior or the medial approach; Cummings et al., Yamada et al., Kiely et al., and Ergin et al. showed higher rates of AVN, which was reported in 86 (46.5%), 35 (30.4%), 7 (14.3%), and 18 (25.7%) hips respectively (Table 3) [23–25, 31]. This can be explained as capsulotomy carries a major risk for avascular necrosis (AVN), and it may be due to the relatively longer follow-up in open reduction series as AVN may be encountered as one of the late sequelae [10–12].

**Table 3:** Comparison of the data of Open reduction studies

Study	Procedure	Sample size		Age (months)		Follow-up (years)		Acetabular index (^o^)		Clinical grading	Radiographic grading	Complications		
		Cases (n)	Hips (n)	Mean	Range	Mean	Range	Pre	Post			AVN	Re-dislocation	Other complications
Cummings et al. [23]	Open reduction through anterior or medial approach +/- pelvic and/or femoral shortening	149	185	23.14	1-121	4.3	2–5	38.4 ± 8.2			*Severin grading* Grade I = 13 Grade II = 39 Grade III 102 Grade IV = 22 Grade V = 3 Grade VI = 6	86 (46.5%)		Need for revision in 60 hips (32.4%)
Yamada et al. [24]	Open Reduction through medial Ludloff approach	103	115	8.5	2–26	20.3	15–18				*Severin grading* Grade I = 27 Grade II = 42 Grade III 38 Grade IV = 7 Grade V = 1 Grade VI = 0	35 (30.4%)		Subluxation in 74 hips (64%) Coxa magna in 23 hips (20%)
Kiely et al. [31]	Open reduction through Ferguson medial approach	45	49	12.3	6–23	6.8	4-12.3	37.4 ± 5.9	20.2 ± 5.4		*Severin grading* Grade I = 26 Grade II = 19 Grade III 4 Grade IV = 0 Grade V = 0 Grade VI = 0	7 (14.3%)	3 (6.1%)	Subluxation in one hip (2%) Residual dysplasia in 5 hips (10.2%) Superficial infection in 2 cases (4.1%)
Ergin et al. [25]	Open reduction through anterior approach	28	31	17	7–24	118	24–192	40 ± 5.63	20 ± 7.51	*McKay’s grading* Grade I = 21 Grade II = 3 Grade III = 6 Grade IV = 1	*Severin grading* Grade I = 17 Grade II = 8 Grade III 1 Grade IV = 5 Grade V = 0 Grade VI = 0	10 (32%)	1 (3.2%)	Residual dysplasia in 2 hips (6.5%)
	Open reduction through medial approach	33	39	13	6–24	132	24–209	41.4 ± 5.52	21 ± 7.20	*McKay’s grading* Grade I = 28 Grade II = 4 Grade III = 6 Grade IV = 1	*Severin grading* Grade I = 29 Grade II = 5 Grade III 2 Grade IV = 3 Grade V = 0 Grade VI = 0	8 (20%)	1 (2.6%)	Residual dysplasia in 3 hips (7.7%)

To our knowledge, this is the largest prospective study to be done for arthroscopic assisted DDH surgery, involving 68 hips. The results showed acceptable outcomes regarding Severin score, acetabular index, and complications rate making this method a viable option for future research and to be employed in patients’ treatment.

We observed that the patients had very good ROM of the operated hips after removal of the cast. This is a very interesting finding although we are not sure if this will hold any benefits to the patients in longer follow-up. This is consistent with other studies describing the benefits of arthroscopic surgeries in sports medicine which minimize the unnecessary soft tissue injuries and scarring making the rehabilitation of the patients quicker [32, 33]. The femoral head ossific nucleus had significant growth and ossification after reduction, even before cast removal, particularly in younger individuals. Minimizing surgical trauma and preservation of the capsule and blood supply through this arthroscopic-assisted approach, led to lowering of postoperative AVN rates compared to studies using open reduction and capsulorrhaphy. We used the sub-adductor as a portal to guide femoral head reduction and improve acetabular cavity exposure (Fig. 5).

However, this study had several limitations. First, we couldn’t assess late sequelae due to short duration and limited follow-up period (24 months). The initial operation time was longer, but this decreased with experience and skill mastery, which could greatly impact the operative time in this series. Our study comprised patients of all ages, including older patients (> 2 years) who had pelvic and/or femoral osteotomies, which may have impacted the outcome. Future studies on specific age groups and with longer follow-ups are needed. Using convenience samples led to a non-representative sample, limiting the generalizability of our findings and introducing sampling bias. Additionally, social desirability bias may have had an impact on our study because patients or their caregivers may have provided more favorable results to please medical professionals or researchers. However, this bias is common in patient reported outcome studies. The lack of a comparator group makes it difficult to assess the relative efficacy of our approach compared to other treatment methods. However, the descriptive nature of our case series did not require the presence of a comparator. Although a considerable number of our patients showed good clinical and radiological results, we are unable to claim that arthroscopic-assisted reduction is better or as effective as other methods without a direct comparison. It is necessary to conduct future studies with a comparative design to overcome this limitation and gain a more thorough understanding of the relative advantages and disadvantages of various treatment approaches for DDH. Finally, although is a post-hoc power analysis exceeded 99% which can guide future research, the results should be interpreted with caution.

## Conclusion

The findings of this study suggest that arthroscopic-assisted reduction combined with femoral and/or pelvic osteotomies when necessary is a promising reliable and safe method in the treatment of irreducible cases of DDH in children older than 6 months. The observed clinical and radiographic outcomes are encouraging, with a low rate of complications such as AVN and re-dislocation.

Switching between the sub-adductor and anterolateral portals offers good visualization of the acetabular cavity and ensures adequate removal of soft tissue obstacles that may prevent complete reduction.

Comparison of these outcomes to larger, multicenter, multi-surgeon studies and with the traditional open reduction technique is essential to confirm the efficacy and safety of this technique and to determine its place in the broader treatment algorithm for DDH.

## Data Availability

No datasets were generated or analysed during the current study.

## Abbreviations

AI: Acetabular Index
ASIS: Anterior Superior Iliac Spine
AVN: Avascular Necrosis
DDH: Developmental Dysplasia of the Hip
REC: Research Ethics Committee
ROM: Range of Motion
TAL: Transverse Acetabular Index
